# Pharmacogenomics of Methotrexate Membrane Transport Pathway: Can Clinical Response to Methotrexate in Rheumatoid Arthritis Be Predicted?

**DOI:** 10.3390/ijms160613760

**Published:** 2015-06-16

**Authors:** Aurea Lima, Miguel Bernardes, Rita Azevedo, Rui Medeiros, Vitor Seabra

**Affiliations:** 1 Cooperativa de Ensino Superior Politécnico e Universitário (CESPU), Instituto de Investigação e Formação Avançada em Ciências e Tecnologias da Saúde (IINFACTS), 4585-116 Gandra PRD, Portugal; E-Mail: vitor.seabra@iscsn.cespu.pt; 2 Grupo de Oncologia Molecular & Patologia Viral—Centro de Investigação do Instituto Português de Oncologia do Porto (CI-IPOP), 4200-072 Porto, Portugal; E-Mail: ruimedei@ipoporto.min-saude.pt; 3 Instituto de Ciências Biomédicas Abel Salazar da Universidade do Porto (ICBAS-UP), 4050-313 Porto, Portugal; E-Mail: rita.pereir@hotmail.com; 4 Faculdade de Medicina da Universidade do Porto (FMUP), 4200-319 Porto, Portugal; E-Mail: mbernardes09@gmail.com; 5 Departamento de Reumatologia do Centro Hospitalar de São João, 4200-319 Porto, Portugal; 6 Grupo de Patologia & Terapêutica Experimental do Centro de Investigação do Instituto Português de Oncologia do Porto (CI-IPOP), 4200-072 Porto, Portugal

**Keywords:** ATP-binding cassette, clinical response, genetic risk index, methotrexate, polymorphisms, rheumatoid arthritis, solute carriers, transporters

## Abstract

Background: Methotrexate (MTX) is widely used for rheumatoid arthritis (RA) treatment. Single nucleotide polymorphisms (SNPs) could be used as predictors of patients’ therapeutic outcome variability. Therefore, this study aims to evaluate the influence of SNPs in genes encoding for MTX membrane transport proteins in order to predict clinical response to MTX. Methods: Clinicopathological data from 233 RA patients treated with MTX were collected, clinical response defined, and patients genotyped for 23 SNPs. Genotype and haplotype analyses were performed using multivariate methods and a genetic risk index (GRI) for non-response was created. Results: Increased risk for non-response was associated to *SLC22A11* rs11231809 T carriers; *ABCC1* rs246240 G carriers; *ABCC1* rs3784864 G carriers; CGG haplotype for *ABCC1* rs35592, rs2074087 and rs3784864; and CGG haplotype for *ABCC1* rs35592, rs246240 and rs3784864. GRI demonstrated that patients with Index 3 were 16-fold more likely to be non-responders than those with Index 1. Conclusions: This study revealed that *SLC22A11* and *ABCC1* may be important to identify those patients who will not benefit from MTX treatment, highlighting the relevance in translating these results to clinical practice. However, further validation by independent studies is needed to develop the field of personalized medicine to predict clinical response to MTX treatment.

## 1. Introduction

Rheumatoid arthritis (RA) is a systemic autoimmune disease, characterized by chronic inflammation of multiple peripheral joints, which leads to destruction of cartilage and bone, progressive deformity and severe disability [1]. The worldwide prevalence of RA is relatively consistent ranging from 0.5% to 1.1% and the incidence vary between 20 and 60 cases per 100.000 inhabitants [2]. In Portugal, the prevalence of RA is 0.36% and the incidence is between 20 and 40 cases per 100.000 inhabitants [3,4]. Overall, the incidence of RA is higher in females, with a gender ratio ranging from 2:1 to 3:1, and the peak of disease onset occurring in the fifth decade of life [5,6].

Earlier detection of RA and the prompt institution of effective and aggressive therapeutic are key factors to achieve the disease remission and improve survival rates [7,8,9]. Several studies from controlled and uncontrolled clinical trials have established that methotrexate (MTX), an antifolate, is an effective disease modifying antirheumatic drug (DMARD) [10,11,12]. Once inside the cells, MTX is metabolized into methotrexate polyglutamates (MTXPGs) by a sequential addition of glutamic acid residues via the enzyme folylpolyglutamate synthetase (FPGS) [13,14]. Polyglutamation of MTX enhances the intracellular retention of MTX promoting the inhibition of folate, methionine and adenosine pathways and, the *de novo* synthesis of purines and pyrimidines, which is considered crucial for anti-inflammatory and antiproliferative therapeutic effects of MTX [13,15,16,17]. Nevertheless, gamma-glutamyl hydrolase (GGH) enzyme removes the glutamic acid residues of MTXPGs and, consequently, MTX can be transported out of the cells [13].

The chemical characteristics of MTX, such its structural composition by a polar glutamate side chain, make it dependent of a carrier-mediated transport system to enter and exit cell membranes [18]. The carrier-mediated transport system includes a network of transporters that belong to two major superfamilies: solute carriers (SLC) and ATP-binding cassette (ABC) transporters [19]. These transporters are expressed in several tissues and have a preeminent effect in MTX pharmacokinetics (PK) parameters such as absorption, distribution and/or elimination [20,21,22]. Consequently, MTX carrier-mediated transport system is considered of great importance for its effectiveness and, despite MTX cost-effectiveness, treatment with this drug is not devoid of drawbacks and different clinical response profiles can occur due to interpatient variability [10,23,24,25]. This variability in MTX clinical effects can be a consequence of MTX PK changes and several lines of evidence suggest that patients’ genetic profile may have a significant role in such variability [26,27,28]. Therefore, over the past decade, numerous pharmacogenomics (PGx) studies were undertaken to investigate individual MTX therapeutic outcomes using genetic information from MTX transport pathway [29,30,31]. Since not all patients benefit from specific therapies, there is a strong and unmet need for pretreatment predictions on therapy outcome. Therefore, this work aims to evaluate the influence of single nucleotide polymorphism (SNPs) in genes encoding for MTX membrane transport proteins on the occurrence of non-response to MTX in Portuguese RA patients.

## 2. Results

### 2.1. Studied Population

This study included follow-up data from 233 patients, 196 (84.1%) females and 37 (15.9%) males, with a median age of 52 ± 11.9 years old, of which 32 (13.7%) were current smokers. In this population, median of serum creatinine (SCr) was 8.20 mg/L (4.00–19.80), median of estimated glomerular filtration rate (eGFR) was 82.0 mL/min/1.73 m^2^ (29.00–186.00) and 30 patients (12.9%) presented chronic renal insufficiency (eGFR < 60 mL/min/1.73 m^2^). Considering disease-related variables, mean age of diagnosis was 40.3 ± 13.2 years old, median disease duration was 8.0 years and mean disease activity score (DAS28) was 4.2 ± 1.3. Only 146 patients (62.7%) used MTX as unique DMARD, while 59 patients (25.3%) were treated with MTX combined with other classic DMARDs (azathioprine, cyclosporine, gold salts, hydroxychloroquine, leflunomide and/or sulphasalazine) and 28 patients (12.0%) were treated with MTX combined with biological DMARDs (adalimumab, etanercept, infliximab, abatacept, anakinra, rituximab and/or tocilizumab). Median MTX treatment duration was 28.0 months with a median dose of 15.0 mg/week (range 2.5–25.0). MTX was administered in 201 patients (86.3%) by *per os* route while in 32 patients (13.7%) was administered by subcutaneous route. Non-response to MTX was observed in 128 (54.9%) patients, of which 107 patients used MTX as unique DMARD.

### 2.2. Genotypes and Haplotypes Characteristics

*SLC22A6* rs11568626 C>T (CC: 232 patients; CT: 1 patient; TT: 0 patients) and *ABCG2* rs2231142 C>A (CC: 200 patients; CA: 33 patients; AA: 0 patients) were excluded from analyses since minor allele frequency was less than 10.0%, and *ABCC1* rs2230671 C>G (CC: 117 patients; CG: 9 patients; GG: 0 patients) was excluded from analyses because genotyping call rates were less than 95.0%. Taking this into account, twenty SNPs were considered. For *ABCB1* rs2032582, three patients (1.3%) presented AG genotype and three patients (1.3%) presented AT genotype. Given the low frequency, these six patients were excluded from the analysis of this SNP. Genotypes distribution was in Hardy–Weinberg Equilibrium HWE (*p* > 0.050) except for the *SLC19A1* rs1051266. SNPs in *SLC16A7*, *SLC19A1*, *ABCB1*, *ABCC1*, *ABCC2* and *ABCG2* were in linkage disequilibrium (LD) (*D′* coefficients mean of 0.81 and range from 0.26 to 1.00; *p* < 0.001), except for *ABCC1* rs246240 and *ABCC1* rs2074087 (*D′* = 0.02; *p* = 0.690).

### 2.3. Genotype Approach and Clinical Response to Methotrexate (MTX)

Table 1 represents the relation between clinical response to MTX and SNPs in *SLCs* and *ABCs*. Regarding SNPs in *SLCs*, our results demonstrated that *SLC22A11* rs11231809 T carriers (*p* = 0.031, odds ratio (OR) = 5.37) were significantly associated with more than five-fold increased risk for non-response to MTX. Regarding SNPs in *ABCs*, *ABCC1* rs246240 G carriers (*p* = 0.008, OR = 5.47) and *ABCC1* rs3784864 G carriers (*p* = 0.015, OR = 4.24) were associated with MTX non-response.

### 2.4. Haplotype Approach and Clinical Response to MTX

Table 2 represents the relation between MTX transporters genes haplotypes and clinical response to MTX. Since *ABCC1* A>G (rs246240) and *ABCC1* G>C (rs2074087) were not in LD, analyses were performed considering the following haplotypes combination: (1) *ABCC1* rs35592, rs246240 and rs3784864; and (2) *ABCC1* rs35592, rs2074087 and rs3784864. Results showed that CGG haplotype for *ABCC1* rs35592, rs2074087 and rs3784864 was statistically significant for non-response compared to TGA haplotype (*p* = 0.025, OR = 4.12). In addition, CGG haplotype for *ABCC1* rs35592, rs246240 and rs3784864 was associated with non-response compared to TAA haplotype (*p* = 0.010, OR = 7.26).

**Table 1:** Relation between single nucleotide polymorphisms (SNPs) in methotrexate (MTX) transporters genes and clinical response to MTX.

*SLCs*	Alleles	Response	Non-Response	*p*	OR (95% CI)	ABCs	Alleles	Response	Non-Response	*p*	OR (95% CI)
*SLC16A7 *A>T (rs3763980)	A carriers	96 (45.5)	115 (54.5)	0.061	Reference	*ABCB1* C>T (rs1045642)	C carriers	79 (45.9)	93 (54.1)	0.255	Reference
	TT	9 (40.9)	13 (59.1)		0.26 (0.06–1.06)		TT	26 (42.6)	35 (57.4)		1.88 (0.63–5.55)
	AA	53 (44.5)	66 (55.5)	0.890	Reference		CC	29 (46.0)	34 (54.0)	0.622	Reference
	T carriers	52 (45.6)	62 (54.4)		0.94 (0.38–2.32)		T carriers	76 (44.7)	94 (55.3)		1.29 (0.47–3.50)
*SLC16A7 *T>G (rs10877333)	T carriers	104 (45.4)	125 (54.6)	0.999	Reference	*ABCB1* C>T (rs1128503)	C carriers	81 (44.0)	103 (56.0)	0.496	Reference
	GG	1 (25.0)	3 (75.0)		1.00 (0.00–0.00)		TT	24 (49.0)	25 (51.0)		1.56 (0.43–5.57)
	TT	72 (44.7)	89 (55.3)	0.738	Reference		CC	35 (45.5)	42 (54.5)	0.244	Reference
	G carriers	33 (45.8)	39 (54.2)		1.19 (0.43–3.33)		T carriers	70 (44.9)	86 (55.1)		1.80 (0.67–4.87)
*SLC19A1* G>A (rs7499)	G carriers	94 (48.2)	101 (51.8)	0.851	Reference	*ABCB1* G>A/T (rs2032582)	G carriers	80 (44.4)	100 (55.6)	0.706	Reference
	AA	11 (28.9)	27 (71.1)		1.14 (0.28–4.58)		TT	23 (48.9)	24 (51.1)		1.27 (0.36–4.47)
	GG	47 (51.6)	44 (48.4)	0.613	Reference		GG	36 (44.4)	45 (55.6)	0.349	Reference
	A carriers	58 (40.8)	84 (59.2)		1.28 (0.49-3.30)		T carriers	67 (45.9)	79 (54.1)		1.62 (0.59–4.44)
*SLC19A1* G>A (rs1051266)	G carriers	80 (46.8)	91 (53.2)	0.924	Reference	*ABCC1* T>C (rs35592)	T carriers	95 (45.2)	115 (54.8)	0.630	Reference
	AA	25 (40.3)	37 (59.7)		1.05 (0.36–3.09)		CC	10 (43.5)	13 (56.5)		1.47 (0.31–7.09)
	GG	37 (46.2)	43 (53.8)	0.672	Reference		TT	56 (45.2)	68 (54.8)	0.130	Reference
	A carriers	68 (44.4)	85 (55.6)		1.23 (0.47–3.18)		C carriers	49 (45.0)	60 (55.0)		2.12 (0.80–5.58)
*SLC19A1* A>G (rs2838956)	A carriers	91 (47.6)	100 (52.4)	0.512	Reference	*ABCC1* A>G (rs246240)	A carriers	102 (45.3)	123 (54.7)	0.846	Reference
	GG	14 (33.3)	28 (66.7)		1.61 (0.39–6.66)		GG	3 (37.5)	5 (62.5)		0.76 (0.05–11.46)
	AA	39 (47.6)	43 (52.4)	0.813	Reference		AA	73 (45.9)	86 (54.1)	0.008 *	Reference
	G carriers	66 (43.7)	85 (56.3)		0.89 (0.33–2.39)		G carriers	32 (43.2)	42 (56.8)		5.47 (1.56–19.25)
*SLC19A1 *G>A (rs3788200)	G carriers	90 (47.1)	101 (52.9)	0.504	Reference	*ABCC1 *G>C (rs2074087)	G carriers	101 (45.5)	121 (54.5)	0.419	Reference
	AA	15 (35.7)	27 (64.3)		1.62 (0.39–6.68)		CC	4 (36.4)	7 (63.6)		0.42 (0.05–3.42)
	GG	41 (50.0)	41 (50.0)	0.285	Reference		GG	62 (42.5)	84 (57.5)	0.104	Reference
	A carriers	64 (42.4)	87 (57.6)		1.69 (0.65–4.42)		C carriers	43 (49.4)	44 (50.6)		0.46 (0.18–1.18)
*SLC22A11* T>A (rs11231809)	T carriers	86 (43.0)	114 (57.0)	0.031 *	Reference	*ABCC1* G>A (rs3784864)	G carriers	76 (42.7)	102 (57.3)	0.015 *	Reference
	AA	19 (57.6)	14 (42.4)		0.19 (0.04–0.86) ^(a)^		AA	29 (52.7)	26 (47.3)		0.24 (0.07–0.76) ^(b)^
	TT	29 (36.2)	51 (63.8)	0.116	Reference		GG	31 (46.3)	36 (53.7)	0.402	Reference
	A carriers	76 (49.7)	77 (50.3)		0.44 (0.16–1.22)		A carriers	74 (44.6)	92 (55.4)		0.64 (0.23–1.80)
*SLC46A1* G>A (rs2239907)	G carriers	89 (47.8)	97 (52.2)	0.429	Reference	*ABCC2 *G>A (rs717620)	G carriers	102 (45.1)	124 (54.9)	0.486	Reference
	AA	16 (34.0)	31 (66.0)		1.61 (0.49–5.28)		AA	3 (42.9)	4 (57.1)		0.30 (0.01–8.89)
	GG	42 (48.3)	45 (51.7)	0.986	Reference		GG	59 (43.7)	76 (56.3)	0.576	Reference
	A carriers	63 (43.2)	83 (56.8)		1.01 (0.39–2.61)		A carriers	46 (46.9)	52 (53.1)		0.77 (0.31–1.91)
*SLCO1B1* T>C (rs4149056)	T carriers	86 (46.5)	99 (53.5)	0.812	Reference	*ABCC2* C>T (rs4148396)	C carriers	81 (44.0)	103 (56.0)	0.677	Reference
	CC	19 (39.6)	29 (60.4)		0.87 (0.28–2.69)		TT	24 (49.0)	25 (51.0)		0.78 (0.25–2.45)
	TT	82 (48.5)	87 (51.5)	0.935	Reference		CC	29 (38.7)	46 (61.3)	0.265	Reference
	C carriers	23 (35.9)	41 (64.1)		1.05 (0.36–3.07)		T carriers	76 (48.1)	82 (51.9)		0.55 (0.20-1.56)
						*ABCG2* T>C (rs13120400)	T carriers	95 (44.8)	117 (55.2)	0.188	Reference
							CC	10 (47.6)	11 (52.4)		0.36 (0.08–1.65)
							TT	52 (44.8)	64 (55.2)	0.226	Reference
							C carriers	53 (45.3)	64 (54.7)		1.84 (0.68–4.96)
						*ABCG2* G>A (rs17731538)	G carriers	101 (45.1)	123 (54.9)	0.898	Reference
							AA	4 (44.4)	5 (55.6)		1.16 (0.13–10.55)
							GG	63 (46.0)	74 (54.0)	0.994	Reference
							A carriers	42 (43.8)	54 (56.2)		1.00 (0.39–2.55)

* *p* value <0.05 is considered to be of statistically significance. *p* value, odds ratio (OR) and 95% confidence intervals (CI) corresponds to multivariate logistic regression adjusted to patient-related variables (gender, age, smoking, eGFR and SCr), disease-related variables (diagnosis age and disease duration), and treatment-related variables (folic acid, corticosteroids, NSAIDs, other concomitant DMARDs and MTX administration characteristics such as dose, treatment duration and administration route); ^(a)^ When reference was AA genotype: OR = 5.37, 95% CI: 1.17–24.71; ^(b)^ When reference was AA genotype: OR = 4.24, 95% CI: 1.32–13.65; A: adenine; C: cytosine; CI: confidence interval; DMARDs: disease modifying antirheumatic drugs; eGFR: estimated glomerular filtration rate; G: guanine; MTX: methotrexate; NSAIDs: non-steroidal anti-inflammatory drugs; OR: odds ratio; SLC: solute carrier; SLCO: solute carrier organic anion transporter; SCr: serum creatinine concentration; SNP: single nucleotide polymorphism; T: thymine.

**Table 2:** Relation between MTX transporters genes haplotypes and clinical response to MTX.

Haplotype				Estimated Frequency (%)	*p*	OR (95% CI)
***SLC16A7* A>T (rs3763980)**	***SLC16A7* T>G (rs10877333)**					
A	T			54.5		Reference
T	T			29.2	0.360	0.72 (0.36–1.44)
A	G			16.3	0.890	1.11 (0.38–3.06)
***SLC19A1* G>A (rs7499)**	***SLC19A1* G>A (rs1051266)**	***SLC19A1* A>G (rs2838956)**	***SLC19A1* G>A (rs3788200)**			
G	G	A	G	48.7		Reference
A	A	G	A	33.6	0.430	1.38 (0.62–3.03)
G	A	A	G	5.8	0.860	0.88 (0.21–3.66)
G	A	G	A	4.4	0.830	1.22 (0.19–7.70)
A	G	A	G	2.0	0.330	3.80 (0.26–55.41)
***ABCB1* C>T (rs1045642)**	***ABCB1* C>T (rs1128503)**	***ABCB1* G>A/T (rs2032582)**				
C	C	G		43.7		Reference
T	T	T		37.5	0.470	1.32 (0.63–2.76)
T	C	G		10.7	0.720	0.82 (0.28–2.44)
C	T	T		3.9	0.530	0.49 (0.05–4.55)
C	T	G		2.7	0.820	0.77 (0.08–7.56)
***ABCC1* T>C (rs35592)**	***ABCC1* G>C (rs2074087)**	***ABCC1* G>A (rs3784864)**				
T	G	A		42.6		Reference
C	G	G		18.1	0.025 *	4.12 (1.20–14.09)
T	G	G		17.7	0.061	3.60 (0.95–13.65)
C	C	G		9.4	0.700	0.77 (0.21–2.86)
T	C	G		7.4	0.690	0.72 (0.15–3.54)
***ABCC1* T>C (rs35592)**	***ABCC1* A>G (rs246240)**	***ABCC1* G>A (rs3784864)**				
T	A	A		46.3		Reference
T	A	G		18.9	0.150	2.05 (0.78–5.38)
C	A	G		16.4	0.620	1.26 (0.50–3.16)
C	G	G		11.1	0.010 *	7.26 (1.64–32.14)
T	G	G		6.2	0.370	2.24 (0.39–12.83)
***ABCC2* G>A (rs717620)**	***ABCC2* C>T (rs4148396)**					
G	C			55.3		Reference
A	T			22.2	0.370	0.66 (0.26–1.64)
G	T			22.2	0.390	0.72 (0.34–1.52)
***ABCG2* T>C (rs13120400)**	***ABCG2* G>A (rs17731538)**					
T	G			48.6		Reference
C	G			28.7	0.730	1.15 (0.53–2.49)
T	A			21.7	0.870	1.07 (0.46-2.51)

* *p* value <0.05 is considered to be of statistically significance. *p* value, odds ratio (OR) and 95% confidence intervals (CI) corresponds to multivariate logistic regression adjusted to patient-related variables (gender, age, smoking, eGFR and SCr), disease-related variables (diagnosis age and disease duration), and treatment-related variables (folic acid, corticosteroids, NSAIDs, other concomitant DMARDs and MTX administration characteristics such as dose, treatment duration and administration route); A: adenine; ABC: ATP-binding cassette; C: cytosine; CI: confidence interval; DMARDs: disease modifying antirheumatic drugs; eGFR: estimated glomerular filtration rate; G: guanine; MTX: methotrexate; NSAIDs: non-steroidal anti-inflammatory drugs; OR: odds ratio; SCr: serum creatinine concentration; SLC: solute carrier; T: thymine.

### 2.5. Genetic Risk Index and Clinical Response to MTX

**Figure 1:**
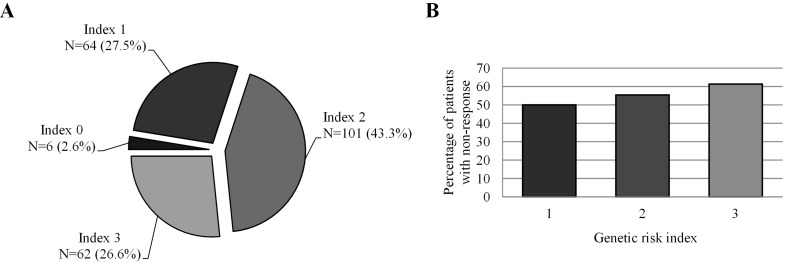
(**A**) Contribution of genetic risk index (GRI) for the occurrence of non-response to MTX; (**B**) Percentage of rheumatoid arthritis (RA) patients with non-response to MTX in relation to GRI.

A genetic risk index (GRI) for non-response to MTX was created for each patient by the sum of risk genotypes from the SNPs that revealed to be statistically significant associated with clinical response to MTX (Table 1). The GRI was adjusted for clinicopathological variables possibly influencing clinical response to MTX as described in Methods section. The risk genotypes were as follow: *SLC22A11* rs11231809 T carriers; *ABCC1* rs246240 G carriers and *ABCC1* rs3784864 G carriers. Figure 1 represents the contribution of GRI for the occurrence of MTX non-response in RA patients. The number (%) of patients is given for each incremental unit of the index.

Index 0 included *SLC22A11* rs11231809 AA + *ABCC1* rs246240 AA + *ABCC1* rs3784864 AA. Index 1 included *SLC22A11* rs11231809 T carriers; *ABCC1* rs246240 G carriers or *ABCC1* rs3784864 G carriers. Index 2 included *SLC22A11* rs11231809 T carriers + *ABCC1* rs246240 G carriers; *SLC22A11* rs11231809 T carriers + *ABCC1* rs3784864 G carriers; *ABCC1* rs246240 G carriers + *ABCC1* rs3784864 G carriers. Index 3 included *SLC22A11* rs11231809 T carriers + *ABCC1* rs246240 G carriers + *ABCC1* rs3784864 G carriers.

The GRI ranged from 0 to 3. Patients with Index 2 were 6.37-fold (95% CI: 1.45–27.99) more likely to be non-responders than those with Index 1 (*p* = 0.014), patients with Index 3 were 9.08-fold (95% CI: 1.54–53.48) more likely to be non-responders compared with those with Index 2 (*p* = 0.015) and patients with Index 3 were 16.06-fold (95% CI: 1.85–139.28) more likely to be non-responders compared with those with Index 1 (*p* = 0.012).

## 3. Discussion

Interpatient variability in clinical response observed in RA patients under MTX treatment may be due to SNPs in genes encoding for MTX membrane transport proteins. This study aims to evaluate the influence of twenty-three SNPs in *SLCs* and *ABCs* MTX transporters as putative predictors of clinical response to MTX in Portuguese RA patients. Hence, we have performed multivariate logistic regression analysis adjusted to potential confounders, particularly to clinicopathological variables, such as patient-, disease- and treatment-related variables, using genotype- and haplotype-based approaches and considering a genetic risk index. This is the first report analyzing the association of SNPs in genes encoding for MTX membrane transport proteins with clinical response to MTX in the Portuguese RA population. In addition, this study is relevant because it overcomes the lack of data regarding the impact of several SNPs in genes involved in the MTX transport pathway in clinical response to MTX while provides a potential pathophysiological explanation to the findings.

The studied population was homogenous relatively to ethnic origin and characteristics were in accordance with other reported studies, in regard to disease gender epidemiology and to the diagnosis age [6,32]. The evaluated outcome (clinical response to MTX) was measured attending to DAS28 and after, at least, six months of MTX treatment, because the maximum improvement to MTX tends to occur only after approximately six months of starting with MTX therapy [11]. Non-response to MTX was registered in 128 (54.9%) patients, which is in accordance to literature, that reports a 40%–60% of patients fail to achieve a good response profile [33].

Genotypes distribution of studied SNPs were in HWE and/or were similar to those previously described for Caucasian populations [16,34,35] and/or in the National Center for Biotechnology Information (NCBI) database.

Multivariate analyses demonstrated that *SLC22A11* rs11231809 T carriers were significantly associated with non-response to MTX. SLC22A11, also known as organic anion transporter 4 (OAT4), is a bidirectional transporter highly expressed at the apical membrane of renal proximal tubular cells [36]. It has been described as having a major role in the tubular reabsorption from urine and secretion of MTX [37,38,39,40]. The rs11231809 occurs in an intronic region of *SLC22A11* at chromosome 11 and is characterized by a substitution of a T for an A [41]. To the best of our knowledge, the impact of rs11231809 in *SLC22A11* expression and function is currently unknown and this is the first report regarding its influence in clinical response to MTX. Nevertheless, previous PGx studies demonstrated the influence of this SNP in PK of diuretic torsemide [42]; no statistically significant risk for renal toxicity was described for the antiretroviral drug tenofovir [43]; and the impact of this SNP in MTX-related overall and gastrointestinal toxicity in RA patients demonstrated non-significant results [19]. Consequently, the influence of rs11231809 in the interplay between tubular reabsorption of MTX and its secretion, and, consequently, in PK and pharmacodynamics (PD) of MTX deserves further investigation.

Regarding *ABCC1*, multivariate genotype analysis demonstrated that G carriers for rs246240 and rs3784864 were associated with non-response to MTX. Moreover, and from haplotypes analysis, our results demonstrated that two haplotypes were associated with an increased risk for non-response to MTX, as follow: CGG haplotype for *ABCC1* rs35592, rs2074087 and rs3784864; and CGG haplotype for *ABCC1* rs35592, rs246240 and rs3784864. ABCC1, also known as multidrug resistance-associated protein 1 (MRP1), is encoded by *ABCC1*, located on chromosome 16p13 [41]. It is a polytopic membrane protein, constituted by seventeen transmembrane regions, present in basolateral plasma membranes of enterocytes [44,45] and has been reported as expressed in cells involved in RA pathophysiology. These include CD3-positive T cells in lymphocytic aggregates, RA synovial tissue macrophages in the intimal lining layer, the synovial sublining, and endothelial cells [46]. The rs35592 is characterized by a substitution of a T to a C; the rs246240 consists in a substitution of an A to a G; the rs2074087 denotes a substitution of a C to a G; and the rs3784864 is characterized by a substitution of a G to an A, all in intronic regions of *ABCC1* [41]. The influence of *ABCC1* studied polymorphisms in ABCC1 function and/or in clinical response to MTX in RA patients is currently unknown. Nevertheless, and since ABCs are mainly efflux transporters, we hypothesize that these genotypes and haplotypes should provide an increased MTX efflux, leading to lower intracellular MTX levels and, consequently, to non-response to MTX.

Other extensively studied SNPs such as *SLC19A1* and *SLCO1B1* variants were not significant for clinical response in this study. This could be explained by the existence of other polymorphisms in these transporters that could counterbalance their functionality or the simultaneous expression of other transporters in target cells that could equalize the influx/efflux ratio. Nevertheless, more and larger studies are necessary to support our results.

The calculation of a GRI was used to improve the impact of studied SNPs in the prediction of non-response to MTX. This GRI demonstrated that patients with Index 3 were 16-fold more likely to be non-responders compared with those with Index 1. This highlights the importance of genotyping the SNPs composing the GRI and the urgency of developing the field of therapy personalization for the prediction of non-response to MTX.

Besides the potential importance of our results, the possible study limitations such as the sample size, study design, and a lack of adjustment of significant results for multiple testing in order to minimize false positive results should be emphasized. Since we analyzed twenty SNPs, a Bonferroni-corrected *p*-value of 0.0025 would have to be used as threshold for significance. Consequently, none of the SNPs would have remained statistically significant after adjustment [47]. Given the conservative nature of Bonferroni method and implied constraints from its usage [48], in order to minimize potential false positive results, a multivariate analysis adjusted to variables that could influence the measured outcome was performed. In addition, results from haplotype analysis were concordant to the associations obtained in genotype analysis, thus it is unlikely that the reported significant results were false positives. Beyond the multivariate analyses, other strengths of this study can be highlighted such: (1) our population is relatively homogenous regarding ethnic origin and is representative of clinical cohorts of established and well-defined RA patients; (2) patient characteristics were in accordance with other reported studies in regard to gender and age at diagnosis [6,32]; and (3) having studied twenty-three SNPs in genes encoding for MTX membrane transporter proteins, many of which had never been studied before, in both RA and Caucasian populations. As future perspectives, due to the lack of studies analyzing the expression of MTX transporters in cells involved in the pathophysiology of RA and the impact of SNPs in the function of such transporters, clinical response and/or MTX circulating levels, further evidence is necessary, to support the interpretation of our results. Furthermore, it is important to consider if enterohepatic recirculation contributes to major differences in bioavailability, to reinforce the importance of understanding the role of the SNPs in transporters on clinical response to MTX. It should be also considered that MTX retention is dependent of MTX polyglutamation levels and, thus, genetic polymorphisms in genes encoding to enzymes involved in MTX polyglutamation process deserve future evaluation. Also, future studies should consider that carrier-mediated mechanisms have been referred to differently depending on MTX dose (low-doses implicates a membrane transport pathway while high-doses implicates both a membrane transport pathway and passive diffusion [49]) and, consequently, may influence MTX pharmacokinetics and the clinical response to MTX. Thus, it is important to address this issue when comparisons are drawn between “low-dose disease models” and “high-dose disease models”. Finally, further studies should consider that a possible synergetic effect between MTX and other DMARDs could influence the associations between genetic polymorphisms and clinical response to MTX [50,51]. Thus, multicenter, prospective studies including RA patients at the diagnosis time and without previously MTX treatment should be conducted to confirm our results.

## 4. Experimental Section

### 4.1. Patients and Study Design

This study was developed as a retrospective study in a cohort of consecutive Caucasian patients (≥18 years) with RA treated with MTX conducted between January 2009 and December 2012 at São João Hospital Center (Porto, Portugal). The Ethics Committee for the Health (Comissão de Ética para a Saúde—CES) had approved this study from an ethical point of view on 19 March 2009, reference 33/2009, and informed written consent was obtained from all patients according to the standards of the Helsinki Declaration. Included patients had to meet the 2010 revised classification criteria of American College of Rheumatology (ACR) and European League Against Rheumatism (EULAR) [52] Patients were excluded of the study if had drug abuse history, recent pregnancy or desire to become pregnant during the study.

Therapeutic strategy: All patients were initially treated with 10 mg *per os*/week of MTX in monotherapy. This dose was increased 5 mg for each three weeks the patients did not meet the EULAR criteria for response, *i.e.*, if presented a Disease Activity Score in 28 joints (DAS28) >3.2. Every 3 months, MTX clinical response was evaluated and therapeutic strategies were defined as follow: (1) first evaluation, if patients have no response or show gastrointestinal toxicity, administration route was changed to subcutaneous; (2) second evaluation, if maximum tolerable dose was used without response, MTX therapy was discontinued or associated with other synthetic DMARD; and (3) third evaluation, in patients without response and other contraindication, therapy was changed by associating a biological DMARD. Folic acid supplementation was prescribed to all patients and its regular compliance was registered. Other concomitant drugs, such as corticosteroids, non-steroidal anti-inflammatory drugs (NSAIDs), and other DMARDs were allowed during the study.

Data collection and variable definition: Patients’ demographics, clinicopathological and treatment characteristics were collected from clinical records. Clinical response was assessed using the DAS28 as described by Prevoo *et al.* [53]. Estimated glomerular filtration rate (eGFR) was calculated using the follow Modification of Diet in Renal Disease (MDRD) equation: 186 × (creatinine/88.4) − 1.154 × (Age) − 0.203 × (0.742 if female) × (1.210 if black), available on http://egfrcalc.renal.org/.

Clinical response definition: Non-response was defined when patients presented a DAS28 > 3.2 in two consecutive evaluations. Therefore, non-response to MTX was defined only after six months of MTX therapy. Response to MTX was defined when patients presented a DAS28 ≤ 3.2.

### 4.2. Single Nucleotide Polymorphisms Selection and Genotyping

A total of twenty-three SNPs in ten genes that codify for MTX membrane transporter proteins were selected based on literature attending to their putative effects on MTX transport function and/or MTX clinical response (Table S1) [35,54,55,56,57,58,59,60,61,62,63,64,65,66,67,68,69,70,71,72,73,74]. Whole blood samples from each patient were obtained with standard venipuncture technique. Genomic DNA was extracted with QIAamp DNA Blood Mini Kit (QIAGEN, Hilden, Germany) according to manufacturer instructions and total genomic DNA was quantified, and its purity analyzed, using the NanoDrop 1000 Spectrophotometer v3.7 (Thermo Scientific, Wilmington DE, USA). Sequenom^®^ Assay Design 3.1 software was used to design the primers and genotyping was performed according to standard Sequenom^®^ iPLEX protocol [75]. Results were manually inspected and verified, using the MassARRAY Typer Analyzer v4.0 software (Sequenom^®^, San Diego, CA, USA). For quality control, 10% of the samples were randomly selected for a second analysis and results were 100% concordant.

### 4.3. Statistical Analysis

Statistical analysis was performed with either IBM^®^ SPSS^®^ Statistics for Windows, Version 20.0 (IBM Corp, Armonk, NY, USA) and SNPStats software [76]. Genotype frequencies were assessed and tested for HWE. SNPs were excluded from analysis when genotyping call rates were less than 95% and when minor allele frequency was less than 10.0%. Multivariate analysis by binary logistic regression was used to identify which genotypes and haplotypes were associated with clinical response to MTX, by adjusting to clinicopathological variables possibly influencing disease state and clinical response to MTX. Such variables were selected based either in literature review and/or clinical significance [77,78,79,80,81] and included: (1) patient-related: age, gender, smoking status and renal function (eGFR and SCr); (2) disease-related: diagnosis age and disease duration; and (3) treatment-related: folic acid, corticosteroids, NSAIDs, other DMARDs and MTX administration characteristics (dose, treatment duration and administration route). The LD between SNPs in the same gene was estimated and expressed as *D′* coefficients. Possible haplotypes were tested for association with clinical response to MTX by taking the most frequent haplotype as reference. A GRI for non-response to MTX was created for each patient. The index included the risk genotypes from the SNPs that revealed to be statistically significant with clinical response to MTX. GRI was tested for association with clinical response to MTX in a multivariate analysis. Results were expressed as OR with 95% confidence intervals (CI) and considering a probability (*p*) value of 5% or less as statistically significant.

## 5. Conclusions

In conclusion, this study revealed that genetic polymorphisms in *SLC22A11* and *ABCC1* could be predictors of clinical response to MTX in Portuguese RA patients. Genotyping patients according to these genetic markers may be helpful to identify which patients will not benefit from MTX treatment, highlighting the relevance of developing the field of personalized medicine. Despite the potential of our findings, translation into clinical practice requires larger and multicentric studies in order to clearly endorse the utility of these SNPs.

## Abbreviations

A: adenine
ACR: American College of Rheumatology
ABC: ATP-binding cassette
C: cytosine
CI: confidence interval
DAS28: Disease Activity Score in 28 joints
DMARD: disease modifying antirheumatic drug
eGFR: estimated glomerular filtration rate
EULAR: European League Against Rheumatism
FPGS: folylpolyglutamate synthetase
G: guanine
GGH: gamma-glutamyl hydrolase
GRI: genetic risk index
HWE: Hardy–Weinberg equilibrium
MRP1: multidrug resistance-associated protein 1
MTX: methotrexate
NSAIDs: non-steroidal anti-inflammatory drugs
OAT4: organic anion transporter 4
OR: odds ratio
PD: pharmacodynamics
PGx: pharmacogenomics
PK: pharmacokinetics
RA: rheumatoid arthritis
SLC: solute carrier
SLCO: solute carrier organic anion transporter
SCr: serum creatinine concentration
SNP: single nucleotide polymorphism
T: thymine

## Supplementary Materials

Supplementary materials can be found at http://www.mdpi.com/1422-0067/16/06/13760/s1.
