# Supplementary material for: Pharmacogenomics of Methotrexate Membrane Transport Pathway: Can Clinical Response to Methotrexate in Rheumatoid Arthritis Be Predicted?
Source: Int J Mol Sci. 2015 Jun 16;16(6):13760–80. doi: 10.3390/ijms160613760 (PMC4490522; doi:10.3390/ijms160613760)
Supplement: Supplementary file 1 [file ijms-16-13760-s001.pdf]

Supplementary Information

Table S1. List of the selected polymorphisms for genotyping, reasons for their choice and which were genotyped in this study.

Protein	Gene	rs	Minor Allele Frequency	Polymorphism Denomination	Polymorphism Localization	Reasons for Choice	Refs.	Described in PharmGKB?	Additional rs Described in PharmKB for the Gene [#]?	Genotyped in this Study?
Monocarboxylate transporter 2	*SLC16A7*	rs10877333	20%	218-T8954G (intron)	ch12:60156046	(B,E)	[34]	No	No	Yes
		rs2711655	40%	361+T1278C (intron)	ch12:60166421	(B,E)	[34]	No		No *
		rs3763980	31%	A1333T (Thr445Ser)	ch12:60173356	(B,C)	[34]	Yes		Yes
Folate transporter 1	*SLC19A1*	rs1051266	29%	G80A (His27Arg)	chr21:45537880	(A–C)	[55,56,82–86]	Yes	Yes, only 3 rs were not included due to the proximity of the polymorphisms chosen	Yes
		rs1131596	43%	T-43C (5′UTR)	chr21:46957916	(A–C)	[86,87]	Yes		No *
		rs12659	50%	3945997A>G (Pro192Pro)	chr21:46951556	(A,E)	[20]	Yes		No *
		rs188530	?	12910392C>T (intron)	chr21:27248521	(A)	[20]	No		No *
		rs2838956	39%	1293+T707C (intron)	chr21:46945024	(A,B)	[55,86]	Yes		Yes
		rs3788200	42%	3951012A>G (intron)	chr21:46956571	(A,E)	[20]	Yes		Yes
		rs7499	37%	G*16A (3′UTR)	chr21:46932328	(A,B)	[55]	Yes		Yes
Solute carrier family 22 member 6	*SLC22A6*	rs11568626	?	G149A (Arg50His)	chr11:62752014	(A,C)	[20,88,89]	Yes	No	Yes
Solute carrier family 22 member 8	*SLC22A8*	rs139472995	?	C270T (3′UTR)	chr11:62760439	(A)	[20]	No	No	No *
		rs45566039	?	C>A (Arg149Ser)	chr11:62999835	(A,C)	[20,90]	No		No *
Solute carrier family 22 member 11	*SLC22A11*	rs11231809	42%	T9608745A (intron)	chr11:64302950	(A,C)	[20,42]	Yes	No	Yes
Proton-coupled folate transporter	*SLC46A1*	rs2239907	47%	A*928G (3′UTR)	chr17:26725744	(A,B)	[20,35,85]	No	No	Yes
Solute carrier organic anion transporter family member 1B1	*SLCO1B1*	rs11045879	16%	1865+T4846C (intron)	chr12:21382619	(A,C)	[20,91]	Yes	Yes, only 3 rs were not included due to the proximity of the polymorphisms chosen	No *
		rs2306283	23%	A388G (Asn130Asp)	chr12:21176804	(A–C)	[5,85, 92,93]	Yes		No *
		rs4149056	15%	T521C (Val173Ala)	chr12:21331549	(A–C)	[85,91,94]	Yes		Yes
		rs56387224	?	A388G (Asn432Asp)	ch12:21355583	(A,C)	[95]	No		No *

Table S1. *Cont.*

Protein	Gene	rs	Minor Allele Frequency	Polymorphism Denomination	Polymorphism Localization	Reasons for Choice	Refs	Described in PharmGKB?	Additional rs Described in PharmKB for the Gene #?	Genotyped in this Study?
Folate receptor α	*FOLR1*	rs7928531	?	T-15C (5′UTR)	chr11:71906694	(A,D)	[96]	No	No	No *
Folate receptor β	*FOLR2*	rs13908	?	A103G (Lys35Glu)	chr11:71929731	(D)	[96]	No	No	No *
Folate receptor γ	*FOLR3*	rs7925545	?	A-1280G (5′UTR)	chr11:71845541	(D)	[96]	No	No	No *
		rs7926987	4%	169-C504G (intron)	chr11:71849503	(D)	[96]	No		No *
		rs7926875	4%	169-C565A (intron)	chr11:71849442	(D)	[96]	No		No *
		rs508088	?	17156451C>T (Leu174Profs)	chr11:71850656	(D)	[96]	No		No *
		rs34970007	?	G594A (Lys198Lys)	chr11:71850731	(D)	[96]	No		No *
Multidrug resistance protein 1	*ABCB1*	rs1045642	44%	C3435T (Ile1145Ile)	chr7:87138645	(A–C)	[45,57,64, 65,84,85,97,98]	Yes	No	Yes
		rs1128503	43%	C1236T (Gly412Gly)	chr7:87179601	(A,B)	[85]	Yes		Yes
		rs2032582	43%	G2677A/T (Ala899Ser/Thr)	chr7:87160618	(A,B)	[57,99]	Yes		Yes
		rs4148737	16%	2212-A372G (intron)	chr7:87541836	(A,E)	[20]	Yes		No
Multidrug resistance-associated protein 1	*ABCC1*	rs2230671	21%	G4002A/C	ch16:16228242	(A,E)	[20]	No	No	Yes
		rs2074087	16%	IVS18-C30G (intrão)	chr16:16184231	(A,E)	[20]	No		Yes
		rs11075291	41%	49-G3198A	chr16:16098475	(A,E)	[20]	No		No *
		rs1967120	31%	489+G409A (intrão)	chr16:16108894	(A,E)	[20]	No		No *
		rs2238476	6%	G1960A (intron)	chr16:16213872	(A,B)	[100]	Yes		No *
		rs28364006	?	A3664G (Thr1337Ala)	chr16:16228249	(A,B)	[100]	Yes		No *
		rs3784864	48%	616-G1641A (intron)	chr16:16125325	(A,B)	[100]	No		Yes
		rs3784862	22%	615+G413A (intron)	chr16:16110891	(A,E)	[100]	Yes		No *
		rs246240	13%	616-A7942G (intron)	chr16:16119024	(A,B)	[100]	Yes		Yes
		rs35592	29%	1219-T176C (intron)	chr16:16141823	(A,B)	[35,98,100]	Yes		Yes

Table S1. *Cont.*

Protein	Gene	rs	Minor Allele Frequency	Polymorphism Denomination	Polymorphism Localization	Reasons for Choice	Refs	Described in PharmGKB?	Additional rs Described in PharmKB for the Gene #?	Genotyped in this Study?
Canalicular multispecific organic anion transporter 1	*ABCC2*	rs717620	19%	C-24T (5′UTR)	chr10:101542578	(A–C)	[35,85,101]	Yes	No	Yes
		rs8187707	5%	C4488T (His1496His)	chr10:101610533	(A)	[20]	Yes		No *
		rs2273697	22%	G1249A (Val417Ile)	chr10:101563815	(A–C)	[85,98,102]	Yes		No *
		rs8187710	5%	G4544A (Cys1515Tyr)	chr10:101611293	(A)	[20]	Yes		No *
		rs4148396	34%	IVS23+T56C (intron)	chr10:101591944	(A,B)	[35,98]	Yes		Yes
		rs3740066	34%	C3972T (Ile1324Ile)	chr10:101604206	(A,E)	[20]	Yes		No *
Canalicular multispecific organic anion transporter 2	*ABCC3*	rs4148416	3%	C3039T (Gly1013Gly)	chr17:50676062	(A)	[20]	Yes	No	No *
		rs9895420	?	T-260A (5′UTR)	chr17:48712038	(A)	[20]	Yes		No *
ATP-binding cassette sub-family G member 2	*ABCG2*	rs2231142	10%	C421A (Gln141Lys)	chr4:89052323	(A–C)	[71,85,98]	Yes	Yes, only 1 rs was not included due to the proximity of the polymorphisms chosen	Yes
		rs17731538	24%	204 −C1592T (intron)	chr4:89055379	(A,B)	[98,100]	Yes		Yes
		rs13120400	32%	1194+A928G (intron)	chr4:89033527	(A,B)	[100]	Yes		Yes

Additional rs described in PharmKB for the gene that were related to methotrexate; * Not genotyped due to incompabilities between SNPs in the same Plex. This problem was a technical limitation of the used Sequenom® MassARRAY iPLEX as genotyping technique; (A) Protein was described as playing a role in methotrexate membrane transport pathway (evidence from literature and PharmGKB database—https://www.pharmgkb.org/); (B) Polymorphism was previously studied regarding its association with methotrexate therapeutic outcome in “low-dose” disease models (evidence from literature); (C) It was previously demonstrated the polymorphism has a functional impact in protein (evidence from literature); (D) Role of the protein in transporting folic acid that possibly has a role in transporting methotrexate; (E) Minor allele frequency adequate for the cohort size. ? Unknown for the European population.
